# Measuring the Normal Stress Distribution Acting on a Locked-Wheel of Push–Pull Locomotion Rovers via a Wheel Sensor System

**DOI:** 10.3390/s20164434

**Published:** 2020-08-08

**Authors:** Daisuke Fujiwara, Tetsuya Oshima, Kojiro Iizuka

**Affiliations:** 1 Department of Functional Control Systems, Shibaura Institute of Technology, Saitama 337-8570, Japan; 2 Department of Machinery and Control Systems, Shibaura Institute of Technology, Saitama 337-8570, Japan; mf18016@shibaura-it.ac.jp (T.O.); iizuka@shibaura-it.ac.jp (K.I.)

**Keywords:** push–pull locomotion, resistance force, wheel sensor system, locked–wheel, loose soil, planetary rover

## Abstract

The resistance force generated when the locked-wheel acts on the soil is critical for deciding the traveling performance of push–pull locomotion. The resistance force depends on the tangential force of the sliding soil wedge beneath the wheel, and the tangential force depends on the forces of the soil and the wheel perpendicular to the tangential direction. Hence, the normal stress distribution of the locked-wheel can affect the resistance force. Previous studies indicated different insights that describe either a uniform or non-uniform shape of the normal stress distribution. The distribution of the locked-wheel still needs to be examined experimentally. This study measured the normal stress distribution using the wheel sensor system, and the variation of the contact area and slip surface beneath the wheel were also observed in PIV analysis. Those results showed that the normal stress distribution was non-uniform along the wheel contact area, and the change of the distribution was confirmed with the change of the contact area and slip surface. Then, the resistance force calculated by a preliminary model based on the measured data was compared with the total resistance force of the wheel measured by a separate sensor. This comparison provided a theoretical consideration for the measured data.

## 1. Introduction

Autonomous traveling for planetary exploration rovers on loose soil requires information about the wheel–soil interaction. Push-pull locomotion is a unique scheme which uses locomotion while the wheels are locked on ground and it supports the repositioning of other wheels, like an inchworm; good traveling performance has been demonstrated on loose soil [1,2,3,4]. Previous studies have investigated its wheel–soil interaction; for example, the wheel–soil interaction model for push–pull locomotion [1], the difference in behavior between conventional traveling and the push–pull scheme [2], the sinking behavior, and the resistance force [5].

The interaction between the locked-wheel and soil, which is a key factor for traveling, has been analyzed. For instance, the investigation of the phenomenon between each locked-wheel and soil for a wheel walking robot provided the relationship between the resistance force and sinkage [5]. That study also indicated that the resistance force increases with increasing sinkage, and the stress distribution under the locked-wheel was uniform along the contact area. The observation of soil flow beneath the locked-wheel while it moved horizontally depicted the shape of soil wedge [6]. The soil flow analysis by particle image velocimetry (PIV) beneath the locked-wheel indicated the different phenomenon between the locked-wheel and normal traveling [2]. The wheel–soil interaction model confirmed non-uniform stress distribution and the difference of forces distribution between the locked–wheel mode and a normal rolling mode for the Marsokhod rover [1].

Furthermore, the investigation for the interaction between a pipeline and seabed provided insights into the contact area, slip surface, and normal stress distribution of the cylindrical objects that act on the soil. The estimation model for the resistance force of the pipeline based on the limit analysis method optimized the slip surface in the soil. This model estimated its resistance force and the normal stress distribution along the slip surface [7]. These results indicated that the normal stress distribution along the slip surface is not uniform. Furthermore, the soil flow simulation beneath the pipeline by the distinct element method (DEM) provided the different types of slip surfaces depending on the different moving directions, and this study confirmed the validation of its shape by comparing it with the observation results of soil flows [8]. Among those studies, there are different views in wheel-working, pipeline, and wheel model for Marsokhod studies regarding the normal stress-distribution. The experimental data of normal stress distribution for the locked-wheel has not been discussed enough. In soil mechanics, the resistance force of the soil wedge is calculated by the sum of the shear force that is generated along the slip surface. The shear force depends on forces from an object and a soil wedge perpendicular to a direction tangential to the slip surface. Understanding the normal stress distribution of the locked-wheel during towing can help to estimate an accurate resistance force.

Measuring forces between the wheel and the ground is a good way to understand vehicle performance. Many studies have tried to measure wheel forces for the various kinds of vehicles [9,10,11]. The wheel forces from loose soil have been measured using a wheel sensor system that has tactile sensors. To understand the complex wheel–soil interaction, this system has measured the stress distribution of the rotating wheel on loose soil [12,13,14,15,16].

This paper, therefore, investigates the normal stress distribution between soil and wheel during towing using the wheel sensor system. Additionally, this paper simultaneously confirms the contact point of the wheel and the slip surface beneath the wheel by PIV analysis. Then, this paper provides a theoretical consideration of those data through the calculation of the resistance force via a preliminary locked-wheel model using experimental data. Those data can provide the knowledge of the normal stress distribution of the locked-wheel and can also advance understanding of the resistance force of the locked-wheel.

## 2. Push–Pull Locomotion Mechanism

**Figure 1:**
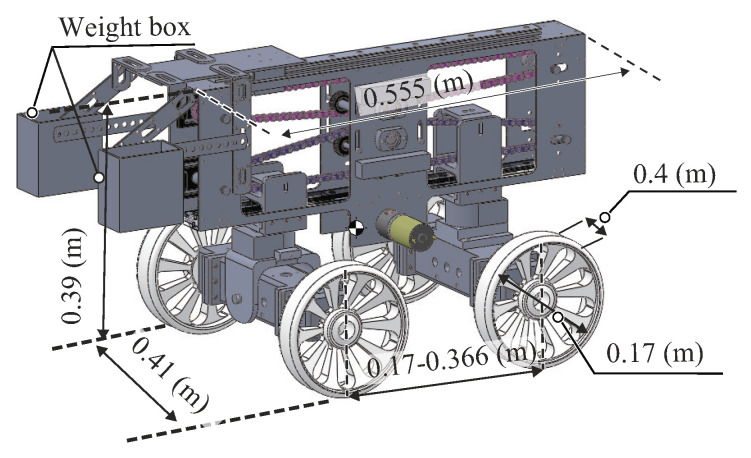
Schematic view of the testbed rover with the function of push–pull locomotion [17].

**Figure 2:**
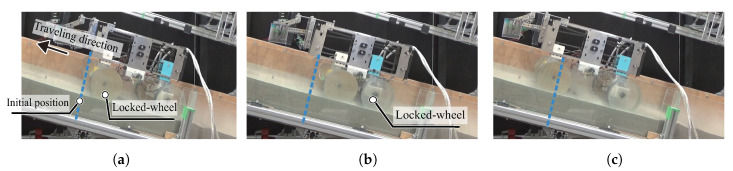
Actual image of traveling experiment on soil: The rover firstly shrinks its wheel-base, and the front wheel is fixed to the ground and supports the rear wheels’ motion (**a**,**b**). Next, the rover extends its wheel-base, and the rear wheel is also fixed to the ground and supports the front wheels’ motion (**b**,**c**).

The testbed rover owned by our laboratory has the function of push–pull locomotion, as shown in Figure 1. This rover can traverse on loose soil on a slope by shrinking or extending its wheel-base, as shown in Figure 2.

The rover firstly fixes the front wheels to the ground at the initial position (Figure 2a). Next, the rover can shrink its wheel-base using the resistance force of the front wheels (Figure 2a,b). In the next stage, the rover also fixes rear wheels to the ground. Then, the rover extends its wheel-base using the resistance of the rear wheels (Figure 2b,c). The rover can move forward by repeating that scheme.

When the required resistance force that supports the other wheels’ motion is small, the necessary amount of soil accumulated behind the wheels is small, whereas when the required force is large, the locked-wheel needs a large amount of accumulated soil behind the wheels.

## 3. Locked-Wheel and Soil-Wheel Interaction

### 3.1. Resistance Force

**Figure 3:**
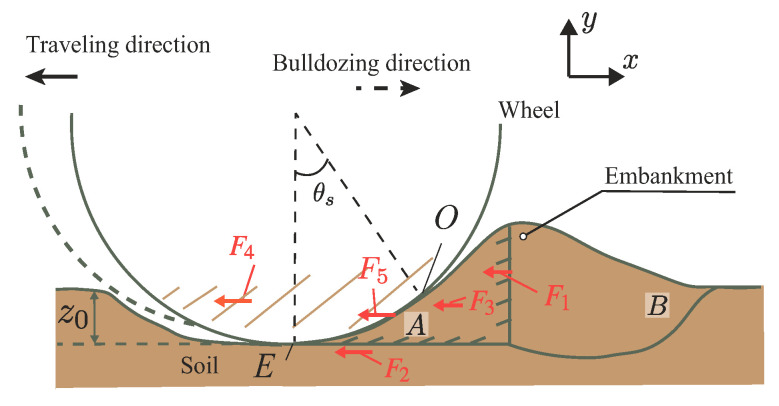
Locked-wheel on the soil surface based on Wong’s observation [6]. The soil wedge (area *A*) beneath the wheel is assumed to move together with the wheel. The surface between soil wedge areas *A* and *B* is assumed as a virtual surface.

A slip surface beneath a wheel is important information to calculate the resistance force of the locked-wheel. Figure 3 shows the soil flow of the locked-wheel observed by Wong [6]. According to this study, when the locked-wheel acts on the soil, a wedge-shaped soil mass (area *A*) is formed in front of the wheel, and it pushes the soil mass area *B*, behaving like a bulldozing blade. $\theta_{s}$ is the soil–wheel contact angle, and *E* and *O* are the entry and exit positions of the contact area. Previous studies also indicated that the shape of the slip surface changes depending on the amount of sinkage and moving direction [7].

As shown in Figure 3, this paper consider that the resistance force mainly comprises five forces as follows:

(1) $$F_{t}=F_{1}+F_{2}+F_{3}+F_{4}+F_{5}+F_{others}$$

where $F_{1}$ is the horizontal component of passive earth force of area *B*, which is calculated as a force of a plate; $F_{2}$ is the horizontal component of the shear force under the soil wedge of area *A*; $F_{3}$ is the horizontal component of the side friction force in area *A*; $F_{4}$ is the horizontal component of the friction force on the side surface of the wheel; $F_{5}$ is the horizontal component of the shear force between wheel surface and soil; and $F_{others}$ indicates the other sundry resistances. In this study, the friction force $F_{4}$ and the shear force $F_{5}$ were neglected in theoretical considerations. This was done because the wheel for the experiment had a smooth side surface. That means that the friction force $F_{4}$ was expected to be relatively small. The weight configuration of the testbed rover was light. Although the shear force $F_{5}$ can be generated, the shear force is expected to be small compared to the force normal to the wheel. Therefore, the shear force $F_{5}$ is not considered theoretically in this paper. The parameter $z_{0}$ indicates the sinkage of the wheel, and it increases depending on an increasing towing distance.

### 3.2. Wheel Sinkage

**Figure 4:**
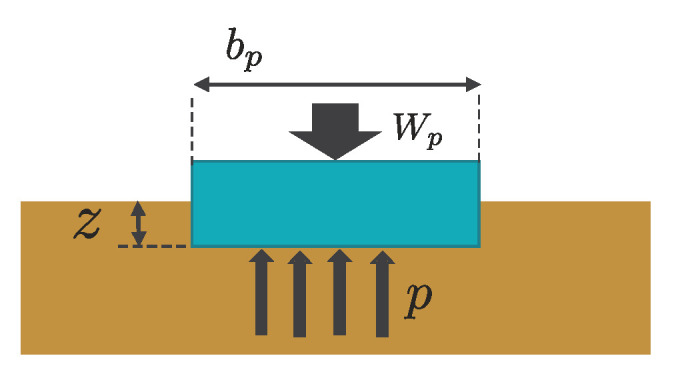
The relationship between a plate and soil.

The sinkage of the static wheel on the loose soil was defined in the Terramechanics field. Bekker [18] defined the pressure–sinkage relationship of a plate, as shown in Figure 4. The normal stress p(z) acting on a plate from soil is as follows:

(2) $$p\left(z\right)=\left(\frac{k_{c}}{b_{p}}+k_{\phi }\right)z^{n}$$

where $k_{c}$, $k_{\phi }$, and *n* are the pressure–sinkage parameters. The parameters $k_{c}$, $k_{\phi }$, and *n* can be obtained by a plate sinkage test of a minimum of two tests with two sizes of plates having different widths [19], and $b_{p}$ indicates the width of a rectangular contact area of a plate.

**Figure 5:**
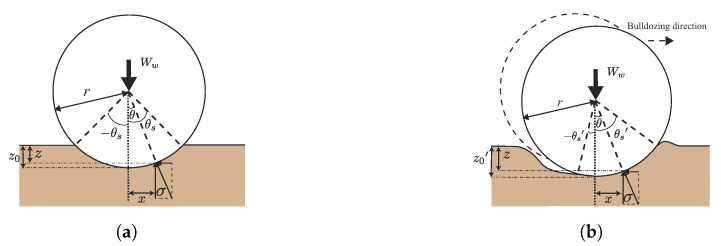
Relationship between sinkage and wheel. (**a**) Wheel sinkage on loose soil. (**b**) Locked-wheel sinkage during bulldozing.

Additionally, Bekker defined the rigid wheel–soil interaction model on loose soil based on the pressure–sinkage model, as shown in Figure 5a. The pressure acts on each wheel surface, as shown in Equation (3).

(3) $$\begin{array}{c} p\left(\theta \right)=\left(\frac{k_{c}}{b_{w}}+k_{\phi }\right)r^{n}{\left(\cos\theta -\cos\theta_{s}\right)}^{n} \end{array}$$

where the θ is the wheel angle. Then, the vertical load of the wheel $W_{w}$ is obtained from Equation (4):

(4) $$\begin{array}{c} \begin{array}{c} W_{w}=b_{w}r\int_{-\theta_{s}}^{\theta_{s}}p\left(\theta \right)\cos\left(\theta \right)d\theta \end{array} \end{array}$$

where $b_{w}$, *r* indicate a wheel width and radius.

When the locked-wheel is towed, the contact area shifts to the towing direction. However, equilibrium (Equation (4)) should be maintained. Therefore, the contact area of the towing side becomes large. Consequently, the wheel sinks into the soil, and sinkage $z_{0}$ increases, as shown in Figure 5.

## 4. Wheel Sensor System

### 4.1. Sensor System

The wheel sensor system which had tactile force sensors on the wheel surface measured the stress distribution of the rotational wheel [12,13,15]. These studies confirmed the difference of the wheel–soil phenomenon between the classical model and experimental data.

**Figure 6:**
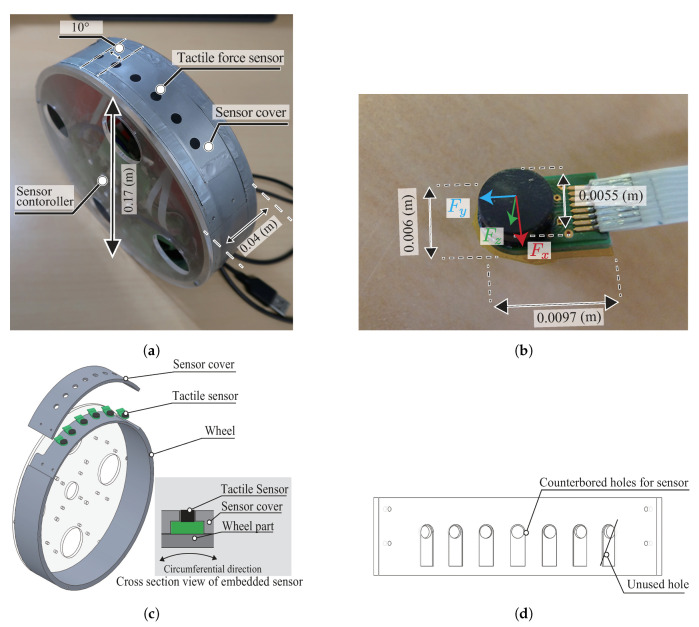
Wheel sensor system. (**a**) Overview of the wheel sensor system. (**b**) Overview of the tactile force sensor. (**c**) Installation of the tactile force sensors on the wheel (**d**) Schematic view of the sensor cover.

For measuring the normal stress distribution and the contact angle between wheel surface and soil, this paper develops the wheel sensor system for a push–pull locomotion rover. Figure 6a shows an overview of the wheel sensor system. Table 1 and Table 2 summarize specifications of the wheel system and tactile force sensor. The wheel size was 0.17 m in diameter and 0.04 m in width. This wheel size was the same as the testbed of push–pull locomotion rover (as mentions earlier in Section 2). The main wheel body was made of aluminum, and the side cover was made of plastic material. This wheel had six tactile sensors Shokac Chip SP (Touchence Inc.), at intervals 10${}^{\circ }$ on the wheel surface, as shown in Figure 6b. The sensor was embedded in the sensor cover, and the cover was installed to the wheel as shown in Figure 6c,d.

One limitation of this research is neglecting the shear force between the wheel surface and the soil. Although the tactile sensor can measure shear force, as shown in Table 2, the shear force of the locked wheel was expected to be smaller than the normal force. The wheel weight was set to be relatively light for our testbed rover’s specifications. Hence, considering the resolution and measurement error of the sensor, measuring the shear force can face the problem of sensor resolution. For this reason, this study neglected the shear force between the wheel and the soil.

The sensor controller board connected to each force sensor via an amplifier board. A logger PC sent the force value from the tactile sensor using logger software.

### 4.2. Sensor Error Analysis

**Figure 7:**
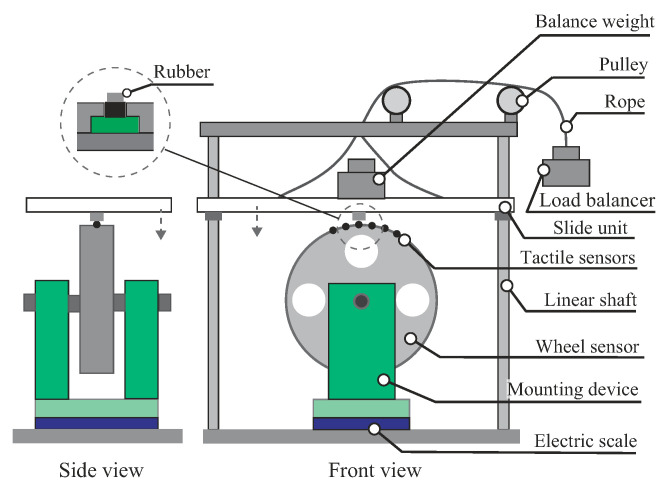
Calibration test setup. The rubber mounted on the slide unit acted perpendicularly on each tactile sensor. The rubber’s area was smaller than the contact area of the sensor. The load balancer offset the weight of the slide unit, and the balance weight was loaded on the slide unit. The mounting device adjusted the rotation angle of the wheel and each sensor was tested. The electric scale measured actual weight, and measured values were compared to each sensor output.

**Figure 8:**
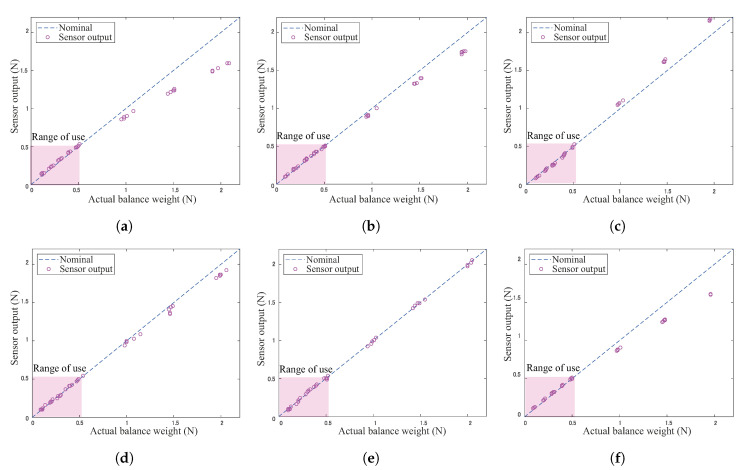
Actual weight vs. sensor output after correction: (**a**) sensor 1, (**b**) sensor 2, (**c**) sensor 3, (**d**) sensor 4, (**e**) sensor 5, (**f**) sensor 6. The most used range for measuring was below 0.5 N.

Each tactile sensor was mounted between the sensor cover and the main body of the wheel, as shown in Figure 6c. When the sensors were assembled on the wheel, the sensor was demonstrated to be sensitive to the counter hole geometry of the sensor cover and the dimensional tolerance of the contact area of the tactile sensor. To address this problem, the calibration test was performed by loading to each tactile sensor in the assembled state. Several balance weights (10–200 g) were loaded on each sensor using the calibration apparatus shown in Figure 7. The sensor calibration confirmed the error between the actual weight (Fa) and each sensor output (Fs), and the coefficient value ks=Fs/Fa was determined. Figure 8 shows the typical results of the sensor outputs through correction. The measuring range was below 1 N, and the most used area for measuring force was below 0.5 N. As seen in Figure 8, the sensor output remained linear and indicates a relatively small error between nominal and output values, especially below 1 N.

## 5. Experiment

### 5.1. Experimental Setup

**Figure 9:**
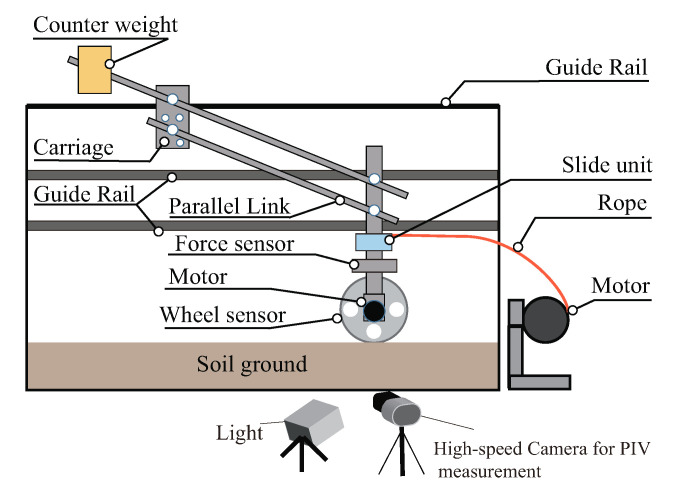
Single wheel tester.

We developed the single wheel tester shown in Figure 9 to measure and confirm the normal stress distribution, the contact angle, and the slip surface during the towing of the wheel. This system comprised the wheel sensor with the motor, parallel link, soil bin, force sensor, and towing motor. The wheel unit was connected to the slide unit and towing guide rails. The wheel unit could move to the vertical direction freely, and the towing did not affect that motion. The parallel link adjusted the wheel weight. The wheel weighed 24.5 N (2.5 kg)—the same as a single wheel of the testbed rover (Figure 1). The soil bin’s width, length, and height were 0.3, 1.2, and 0.18–0.2 m, respectively, and silica sand number 5 filled this area. The towing motor towed the wheel unit at constant speed of 0.0003.41 m/s by PID control. The separate force sensor above the wheel measured the total resistance force acting on the wheel. The motion capture system measured the displacement and sinkage of the wheel. The sampling frequency of all equipment was set to 100 Hz. To observe soil flow beneath the wheel, the high-speed camera recorded the side-cross section view of the soil bin. Then, the PIV software analyzed the images. The experimental procedure was as follows:

- A leveling plate with spikes stirred up the soil at first. Then, the leveling plate smoothed the soil surface without compaction along the sidewall of the soil box.
- The wheel was slowly and carefully set on the soil surface.
- The rope towed the wheel unit at each constant speed.

### 5.2. Measurement Method

**Figure 10:**
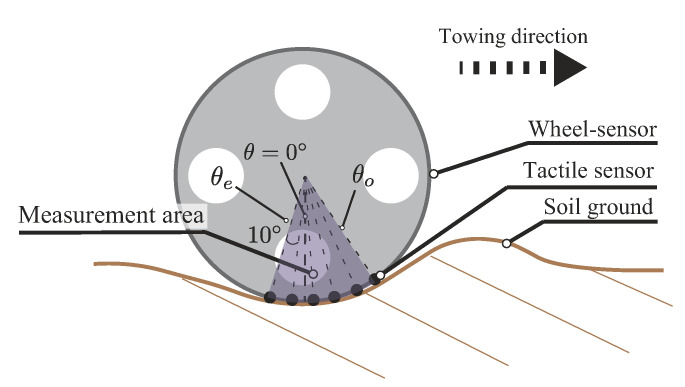
Schematic view of typical sensor position. The position $\theta_{e}$ is the sensor position in the opposite direction to the towing, and $\theta_{o}$ is the sensor position in the towing direction.

**Figure 11:**
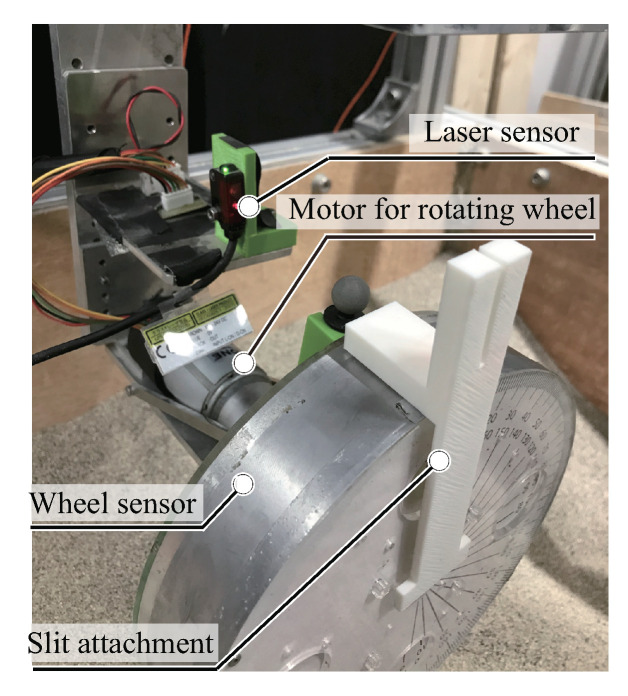
Overview of wheel sensor unit with a laser sensor.

The measurement area of the wheel was 50${}^{\circ }$ (from $\theta_{e}$ to $\theta_{o}$) at an interval angle 10${}^{\circ }$ (Figure 10), and this area acted on the soil surface. Adjusting the measurement area and increasing the angular resolution were realized by rotating the wheel and shifting the mounted sensor position.We measured the force at an interval angle of 5${}^{\circ }$ with this method. The rotate angle of the wheel was controlled by the motor and the laser sensor, as shown in Figure 11. The laser sensor detected the initial position of the wheel, and the wheel stopped at the initial position. Then, the wheel rotated until the target position and measured the required area. We performed four cases of the sensor positions for measuring normal stress distribution, as shown in Table 3. Experimental trials were carried out 5–10 times in each case.

## 6. Result

### 6.1. Raw Data

**Figure 12:**
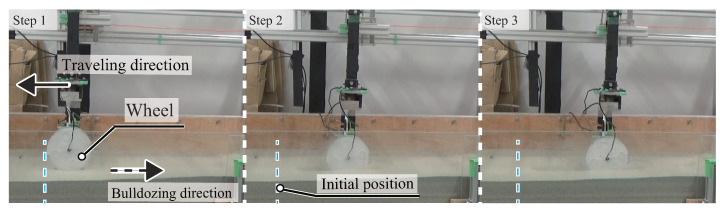
Actual image of wheel towing experiment. Diameter 0.17 m; width 0.04 m; mass 2.5 kg. The wheel was carefully set on the loose soil (step 1). Then, the rope towed the wheel unit (steps 2 and 3).

**Figure 13:**
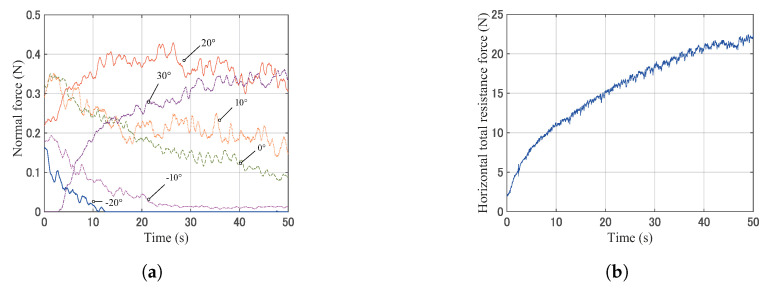

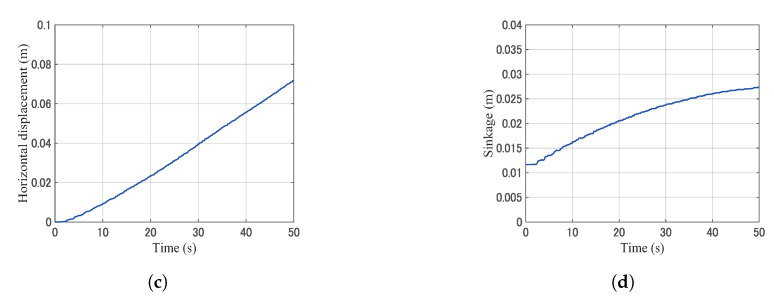
Raw data over time. (**a**) Typical raw data of normal force values at the contact area from −20${}^{\circ }$ to 30${}^{\circ }$ (Case B). (**b**) Total resistance force over time. The data were measured by the separate force sensor installed above the wheel unit (Figure 9). (**c**) Horizontal displacement over time. The data were measured by a motion-capture system. (**d**) Sinkage over time. The data were measured by a motion-capture system.

Figure 12 shows the actual image of the typical experiment. Figure 13 shows typical raw data of the normal force values, the horizontal total resistance force, the horizontal displacement, and the sinkage over time. From these results, the wheel sinks into the soil with an increasing of towing distance. Furthermore, the force values at the contact angles −20${}^{\circ }$ and −10${}^{\circ }$ decrease and finally get close to 0, whereas the forces of the contact angles 20${}^{\circ }$ and 30${}^{\circ }$ increase. These results indicated that the contact area and normal force distribution were changed to the towing direction. Additionally, the total resistance force that was obtained by the separate sensor above the wheel unit rose with an increasing sinkage.

### 6.2. Normal Stress Distribution and Contact Angle

**Figure 14:**
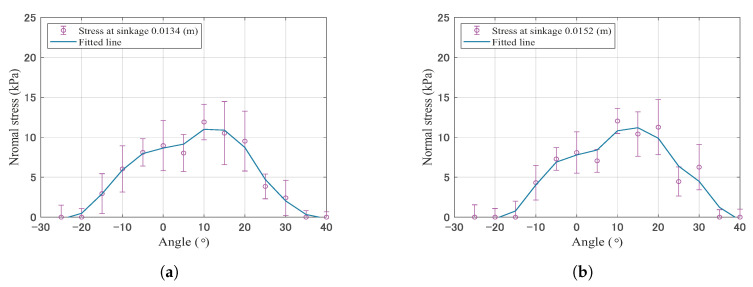

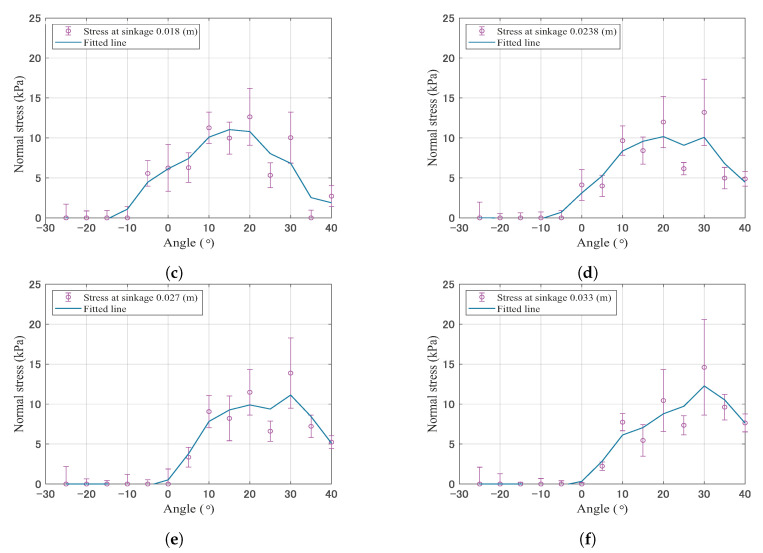
Mean ±SD normal stress at each sinkage and contact angle. Each distribution shows the data when the sensor position $\theta_{e}$ was 0${}^{\circ }$. (**a**) Sinkage 0.013 (m) $\theta_{e}$ = −20${}^{\circ }$, (**b**) Sinkage 0.015 (m) $\theta_{e}$ = −15${}^{\circ }$, (**c**) Sinkage 0.018 (m) $\theta_{e}$ = −10${}^{\circ }$, (**d**) Sinkage 0.0024 (m) $\theta_{e}$ = −5${}^{\circ }$, (**e**) Sinkage 0.027 (m) $\theta_{e}$ = 0${}^{\circ }$, (**f**) Sinkage 0.033 (m) $\theta_{e}$ = 0${}^{\circ }$.

Figure 14 shows the normal stress distribution at each sinkage. Each distribution indicates the distribution when the measured stress of the sensor at the smallest contact angle $\theta_{e}$ became 0. Each datapoint is the average of the 5–10 trials. Table 4 summarizes the smallest contact angle $\theta_{e}$ and sinkage. From this result, we can say that the normal stress distribution was non-uniform along the contact angle; and the shape of the normal stress distribution indicates a concave downward shape. Additionally, the peak position of the distribution shifted to the positive angle with an increasing sinkage, and the smallest contact angles at each sinkage also got close to 0${}^{\circ }$ (directly below the point of the wheel).

Although non-uniform stress distribution still remained, the difference between the collected data at 10, 20, and 30${}^{\circ }$, and 5, 15, and 25 was large, especially in Figure 14d–f. This could have been caused by the measurement method, that is, the rotating wheel with shifting sensor positions. The large resistance force could affect the distortion of the wheel body. When this system faces a large resistance force, this problem needs considering.

### 6.3. Observation Results of Soil Flow by PIV Analysis

**Figure 15:**
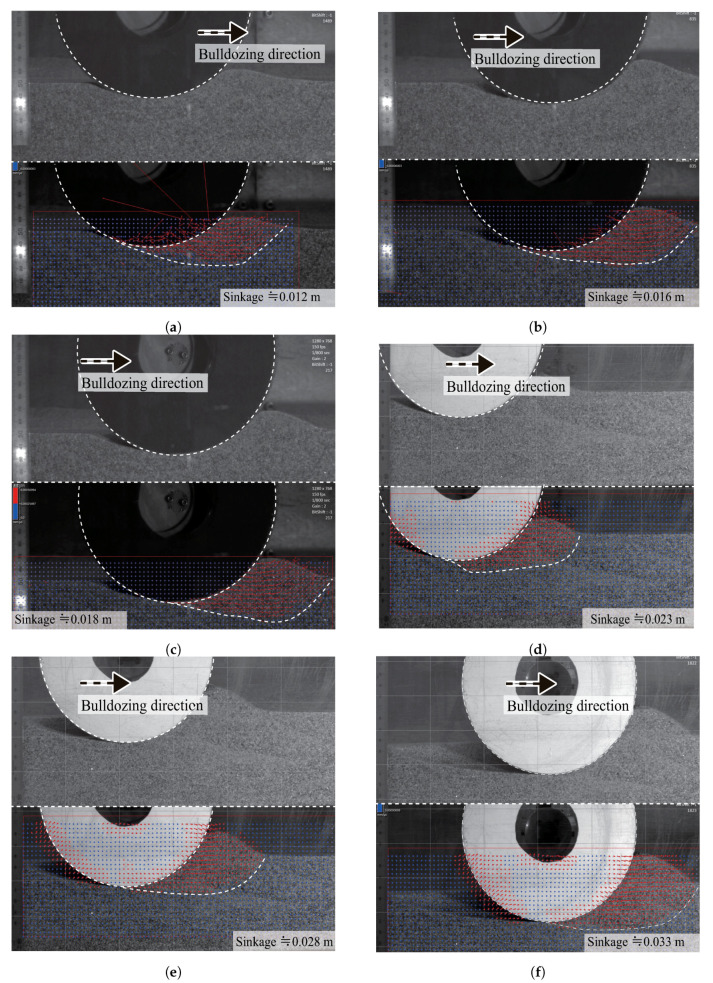
Cross-sectional side view of the soil flow in each sinkage condition. The upper image in each case is the raw image. The bottom images are images after PIV analysis. (**a**) Approximately 0.012 m, (**b**) approximately 0.016 m, (**c**) approximately 0.018 m, (**d**) approximately 0.023 m, (**e**) approximately 0.028 m, (**f**) approximately 0.033 m.

Figure 15 shows a cross-sectional view of the soil flow beneath the locked wheel at different levels of sinkage. Each sinkage condition is approximately the same as the sinkage when the normal stress distribution is measured. The shape of the slip surface appears in the downward direction beneath the wheel due to the sinking wheel at first. Then, the sinking direction of the wheel gradually becomes horizontal.

Additionally, Table 5 summarizes the smallest contact angle at each sinkage degree. This angle was measured by ImageJ software [20]. The smallest contact angle shifted from a negative value to 0${}^{\circ }$ (directly below the point of the wheel).

## 7. Discussion

### 7.1. Normal Stress Distribution

The normal stress distribution of the locked wheel on the dry, loose soil was assumed constant along the contact area [5], whereas it was not constant in the study of the wheel model for Marsokhod rover or the pipeline–soil interaction [1,7]. The knowledge of the experimentally normal stress distribution for the locked-wheel on dry sand has not been discussed enough. The stress distribution measured by the wheel sensor confirmed that the shape of the normal stress distribution changed depending on the sinking direction during towing. It was mountain-shaped, and the peak value was located directly below the wheel at the initial towing stage. Then, the peak value shifted to the towing direction with an increasing sinkage.

Furthermore, PIV analysis confirmed that the slip surface also changed with increasing sinkage. That is, the stress along the slip surface is complex and different depending on the position of the surface, especially at the initial stage of towing. The shear stress along the slip surface was calculated based on the stress perpendicular to the tangential direction. Stress distribution and slip surface can affect the resistance force of the wheel.

### 7.2. Contact Angle

The measured normal stress distribution by the wheel sensor confirmed that the smallest contact angle was the negative value at the initial towing stage. Then, it got close to $0^{\circ }$. This result confirms that the shape of the soil wedge area *A* in the observation result of Wong study [6] (Figure 3) changes depending on the sinkage. Additionally, the same tendency was confirmed by PIV analysis. Furthermore, this tendency corresponds with the DEM analysis of the resistance force for the pipeline on seabed [7,8].

PIV analysis included assuming limiting glass-wall friction and the effect of non-uniform stress distribution along the wheel width [14,15]. The normal stress distribution during the rotating wheel was not uniform across the width when the wheel (size: 0.25 m in diameter and 0.1 m in width) was tested [16]. Additionally, the stress distribution of the wheel (size: 0.26 m in diameter and 0.16 m in width) was measured [15], and non-uniform distribution was reported. From this, there was a possibility that the phenomenon of the soil–wheel interaction observed through the glass wall at the wheel edge differed from that of the center of the wheel. However, the difference in the contact angle between the value from the wheel sensor and that from PIV analysis was relatively small from Table 4 and Table 5. The wheel width (0.04 m) in this study was relatively small; therefore, the difference for the locked-wheel between the wheel’s edge and center could have been small.

### 7.3. Calculating Resistance Force Acting on the Wheel through a Preliminary Model Using Measured Data

**Figure 16:**
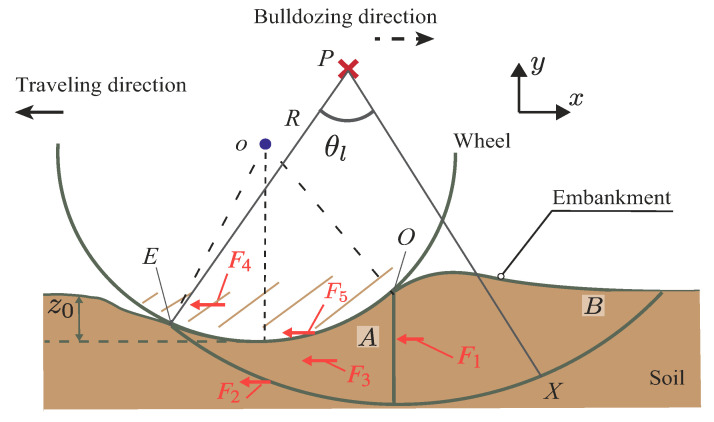
Schematic view of the preliminary model.

Using measured data (normal stress distribution, contact angle, slip surface), we calculated the total resistance force of the locked-wheel through a simple model, as shown in Figure 16. Note that this model is a preliminary model and does not yet include and consider the effect of the shear force between the wheel surface and soil. This is because the weight configuration of the testbed rover in this study was light, and the shear force was expected to be smaller than the normal force. Measuring shear force can face to the problem of sensor resolution, as mentioned earlier in Section 3.1 and Section 4.1. Although the model can underestimate the resistance force, this calculation provides a theoretical consideration for the resistance force of the locked wheel.

The method was based on an observation by Wong [6], as shown in Figure 3. Wong’s observation assumed that soil wedge area A behaved like a plate and acted on soil mass area B. Unfortunately, the shape of soil wedge area A changes depending on the sinking behavior, as shown PIV analysis in Section 6.3, especially in shallow conditions. This study considered this soil wedge variation depending on experimental data and tried to calculate the resistance force. Figure 16 shows the locked-wheel model considering the change of the soil mass shape. This study assumed that a slip surface beneath the wheel functioned as a logarithm spiral. The logarithmic spiral was as shown in Equation (5).

(5) $$\begin{array}{c} \begin{array}{c} PX=R\times e^{\theta_{l}\tan\phi } \end{array} \end{array}$$

where *P* indicates the center of rotation point for the logarithm spiral, *E* indicates the entry contact point of the slip surface, *R* indicates the length of PI, $\theta_{l}$ defines the position of the slip surface, and ϕ indicates the internal friction angle. This shape has often been assumed as a slip surface of the soil wedge in the soil mechanics. To analyze the slope stability of the soil, this shape was optimized by calculating the safety factor, which is a ratio between the force factor of pulling the sliding soil mass downward and the resisting forces against the sliding down of the soil mass in soil mechanics. However, this study confirmed and obtained the contact point, the shape of the slip surface, and the normal stress distribution from the experiments. Hence, this shape was decided on based on the experimental results.

For calculating the resistance force $F_{t}$, this study calculated each horizontal force $F_{1}$, $F_{2}$, and $F_{3}$, except $F_{4}$ and $F_{5}$, as shown in Figure 16. The calculation method was as follows:

#### 7.3.1. Classical Analytical Model for Calculating Force $F_{1}$ against Area *B*

There are several analytical models for predicting the forces of plate tools against soil masses which have been widely studied since the 1960s [21,22,23,24,25,26]. Analytical models have been developed for estimating or calculating the earthmoving, excavation, and cutting forces of a bucket. These models were summarized by [27], and the models have been verified by several types of research [28,29,30]. Furthermore, Scott et al. and Yeomans et al. [31,32] proposed the leg model for planetary rovers and estimated its resistance force using those analytical models.

**Figure 17:**
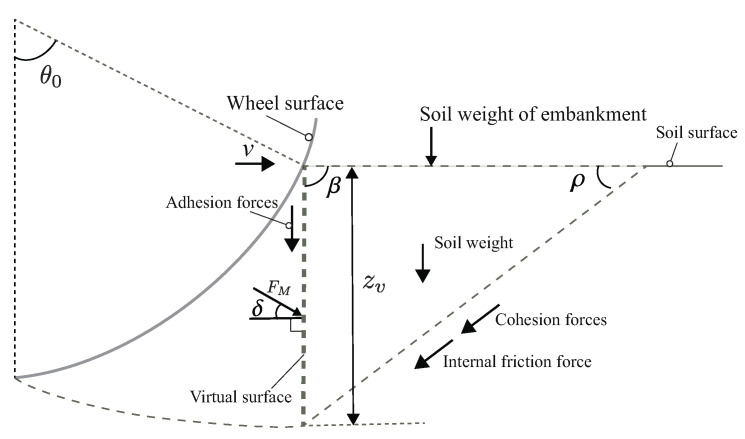
Plate failure plane geometry and variables for force $F_{1}$. The horizontal component of $F_{M}$ is assumed that the force $F_{1}$ from area *B*. The soil wedge beneath the wheel is assumed to move together with the wheel. The surface between soil wedge areas *A* and *B* is assumed as a virtual surface (the length $z_{v}$).

Our previous plate bulldozing tests confirmed that the Mckyes model indicated a value relatively close to that from the experiments in our laboratory environment. Form this result, we chose the Mckyes model for calculating force $F_{1}$ in this study. Figure 17 depicts failure planes and the forces in the analytical models applying for area *B* of the soil flow. The parameters used for the analytical model are shown in the list of notations (Table A1).

#### 7.3.2. Mckyes Model

In 1985, Mckyes et al. [23] proposed the earthmoving model that was first introduced by Reece [33]. The Mckyes model, which can be written as follows, can consider the effects of the soil–tool adhesion, soil–soil cohesion, blade width, surcharge, and inertia. With respect to the failure plane angle ρ, the previous study [34] pointed out that the classical Mckyes model lacks consideration of the failure plane angle ρ depending on the soil heap property in front of a plate. However, this study uses these parameters depending on soil geometry measured by PIV analysis.

(6) $$\begin{array}{c} \begin{array}{c} F_{M}=\left(\frac{b_{w}z_{v}}{\cos(\beta +\delta )+\sin(\beta +\delta )\cot(\rho +\phi )}\right)\times \left[\frac{g\gamma z_{v}}{2}\left(\cot\beta +\cot\rho \right)+q\left(\cot\beta +\cot\rho \right)\right.\\ \left.+c\left(1+\cot\rho \cot\left(\rho +\phi \right)\right)+C_{a}\left(1-\cot\beta \cot\left(\rho +\phi \right)\right)+\frac{\gamma v^{2}\left(\tan\rho +\cot\left(\rho +\phi \right)\right)}{1+\tan\rho \cot\beta }\right] \end{array} \end{array}$$

(7) $$\begin{array}{c} F_{Mh}=F_{M}\sin\left(\beta +\delta \right) \end{array}$$

(8) $$\begin{array}{c} F_{Mv}=F_{M}\cos\left(\beta +\delta \right) \end{array}$$

where $F_{Mh}$ and $F_{Mv}$ represent the horizontal force and vertical force of the total force $F_{M}$ acting to the plate. Herein, force $F_{Mh}$ indicates $F_{1}$ of area *B* (Figure 16).

#### 7.3.3. Shear and Side Friction Force $F_{2}$ and $F_{3}$ of Area *A*

**Figure 18:**
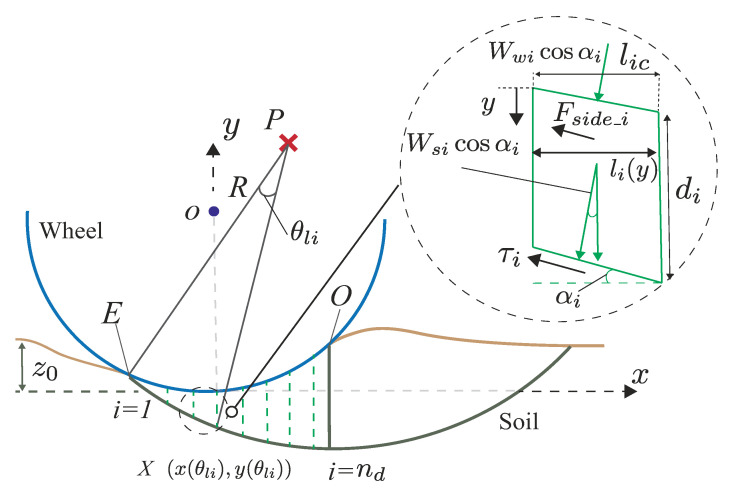
Force acting on each soil wedge beneath the wheel.

The shear strength $F_{2}$ beneath the soil wedge in area *A* was calculated based on Coulomb’s failure criterion, and side friction force $F_{3}$ was calculated based on earth pressure at the rest coefficient. The logarithm spiral, circular line, and assumed vertical line enclose the wedge area A. This paper divides this area into arbitrary sliced blocks. Each force $F_{2}$ and $F_{3}$ was calculated as the sum of the forces that acted on each divided block. Figure 18 shows the wedge area divided into $n_{d}$ blocks and the forces that act on the arbitrary block. $\tau_{i}$ and $F_{side\_i}$ indicate the tangential force and the side friction force at each block. The following equations indicate those forces:

(9) $$\tau_{i}=cb_{w}l_{ic}+\left(W_{si}\cos\alpha_{i}+W_{wi}\cos\alpha_{i}\right)\tan\phi$$

where *c* indicates soil cohesion, $b_{w}$ indicates the wheel width, and $W_{si}$ indicates soil mass weight of the arbitrary block. $W_{wi}$ and $\alpha_{i}$ indicate the vertical component of the normal force calculated from the measured normal stress distribution and the angle between a line tangent to the logarithmic spiral.

Additionally, $F_{side\_i}$ was obtained as follows:

(10) $$F_{side\_i}=\int_{0}^{d_{i}}2\left(c+\sigma_{hi}\tan\phi \right)l_{i}\left(y\right)dy$$

where $d_{i}$ is the depth of each block and $l_{i}\left(y\right)$ is the length of each block at depth y. $\sigma_{hi}$ is the horizontal normal stress of soil, and this is calculated from the vertical normal stress of soil $\sigma_{vi}$ as follows:

(11) $$\begin{array}{cc} \sigma_{hi} & =K_{0}\sigma_{vi}=K_{0}\left(\sigma_{wi}+\gamma gy\right) \end{array}$$

where $K_{0}=1-\sin\phi$ [35] is the coefficient of earth pressure at rest. *y* indicates the depth of the block and $\sigma_{wi}$ is the wheel weight per unit area at each block. The Equation (11) indicates the horizontal normal stress at depth *y*. Following these equations, the forces $F_{2max}$ and $F_{3max}$ were obtained as follows:

(12) $$\begin{array}{c} F_{2max}=\sum_{i=1}^{n}\left(F_{side\_i}\cos\alpha_{i}\right) \end{array}$$

(13) $$\begin{array}{c} F_{3max}=\sum_{i=1}^{n}\tau_{i}\cos\alpha_{i} \end{array}$$

The shear strength $F_{2max}$, $F_{3max}$ can be considered the maximum strength. According to previous research, the available shear strength *H* of soil for a vehicle was proposed by [36]. According to this, the modification of maximum shear strength $H_{0}$ can be considered based on stress–shear displacement relationship as follows:

(14) $$H=H_{0}\left(1-e^{-j/\kappa }\right)$$

where *j* is the shear displacement and κ is the shear deformation modulus. Hence, the shear strength $F_{2}$, $F_{3}$ indicates what follows:

(15) $$F_{2}=F_{2max}\left(1-e^{-j/\kappa }\right)$$

(16) $$F_{3}=F_{3max}\left(1-e^{-j/\kappa }\right)$$

where the *j* is the horizontal displacement of the locked-wheel. For the calculation, the sinkage condition is the same as the sinkage during the normal stress distribution measured in Section 6.2. The calculation uses the experimental contact angle and normal stress distribution as shown in Table 4 and Figure 14. The calculation also uses the slip surfaces observed by PIV analysis, as shown in Figure 15 in Section 6.3. Table 6 summarizes the parameters used in these calculations. The soil deformation modulus κ of silica sand number 5 values ranges between 0.002 and 0.02 m, as reported in [37]. The soil deformation modulus κ values range between 0.01 m (sandy terrain) and 0.025 m (loose sand) according to literature [6]. Given that knowledge, possible values of κ (0.002, 0.02) were used in this paper.

#### 7.3.4. Comparing Calculated Resistance Force and Experimental Results

**Figure 19:**
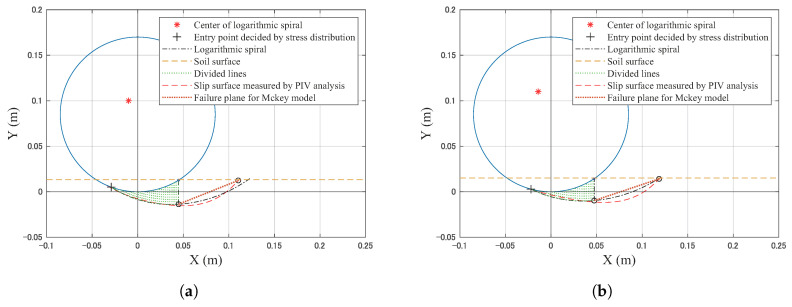

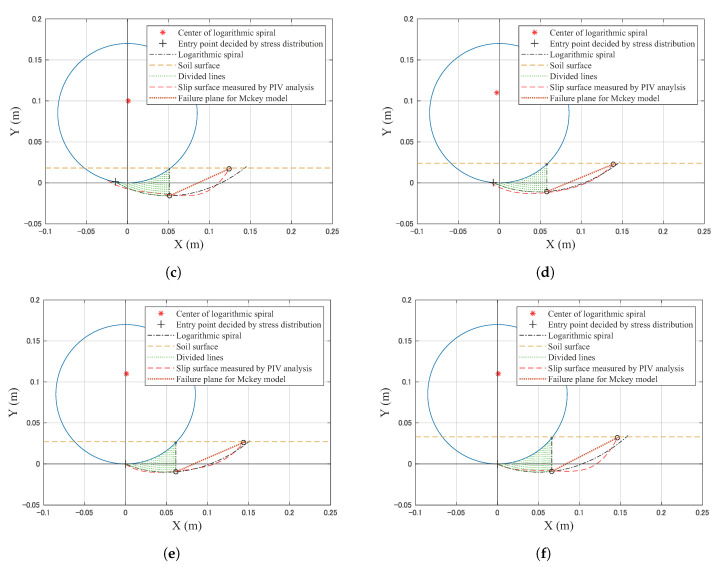
Schematic view of the preliminary model for calculating resistance force. (**a**) sinkage: 0.013 (m), (**b**) sinkage: 0.015 (m), (**c**) sinkage: 0.018 (m), (**d**) sinkage: 0.023 (m), (**e**) sinkage: 0.028 (m), (**f**) sinkage: 0.033 (m).

**Figure 20:**
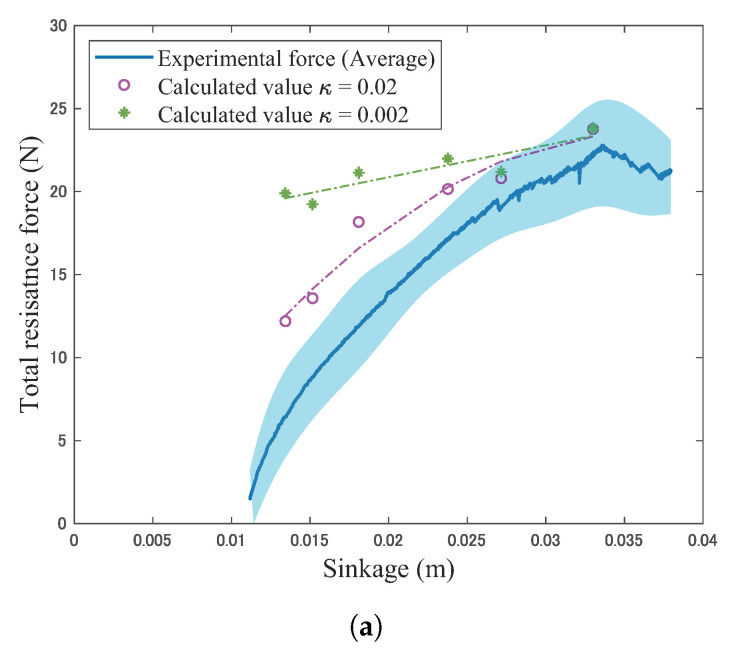

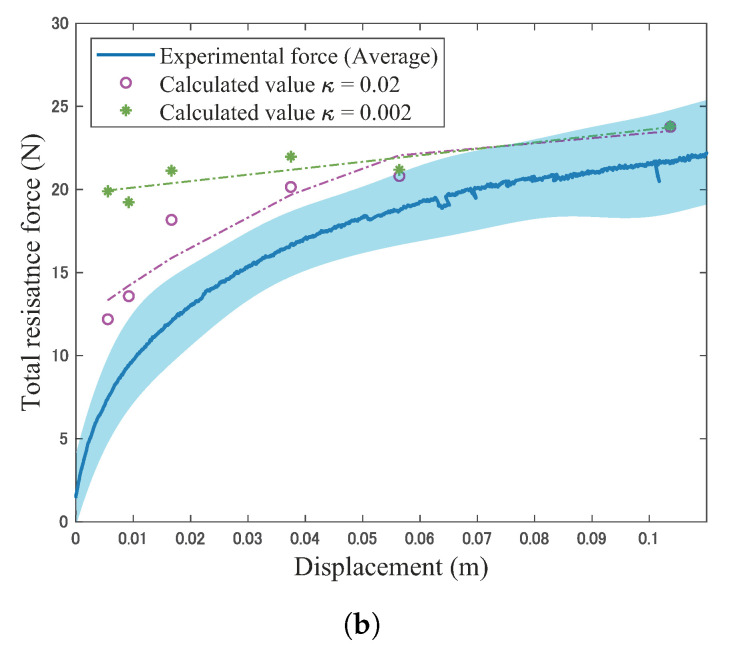
Calculated values that were obtained by the preliminary model vs. total resistance force that was obtained from the separate force sensor above the wheel unit. (**a**) Mean ±SD force vs. sinkage. (**b**) Mean ±SD force vs. displacement.

Figure 19 shows a schematic view of the preliminary model for calculating the resistance force. Each geometry of the slip surface was based on the experimental data. Based on this geometry, the preliminary model calculates the resistance force as shown in Figure 20.

Calculated data indicated a relatively larger value than experimental values, especially under small sinkage and displacement conditions. The calculated resistance force using κ=0.02 captures, relatively, the same trend as the experimental value. The calculated resistance force indicated a relatively close value to the experimental value at large sinkage and displacement. However, the preliminary model did not include the effect of the shear force between the wheel and soil, and the model can underestimate the forces.

## 8. Conclusions

This paper measured the normal stress distribution of the locked-wheel using the wheel sensor system and considered the distribution with the changing of the contact angle and slip surface during towing. Furthermore, we calculated the resistance force of the locked-wheel based on experimental data through the preliminary model, and the calculated values were compared with experimental data as a theoretical consideration. The main conclusions are as follows:

- The normal stress distribution of the locked-wheel was a non-uniform distribution along the contact area. Additionally, the distribution changed depending on sinkage and sinking direction with the changing of the slip surface and contact area. This result differs from the distribution that was investigated for the wheel-working robot [5], whereas the result corresponds with the insight of the wheel–soil interaction for Marsokhod rover and pipeline analysis [1,7].
- The sinking behavior of the locked-wheel made a large change in the normal stress distribution at the initial towing stage as the slip surface changed. Furthermore, the shape of the slip surface spread downward on the wheel, especially at the initial stage. These results suggest that the change of the normal stress distribution can affect the resistance force.
- Although the preliminary model (κ=0.02) indicated relatively the same trend as the experiment, the calculated resistance force indicated relatively large values compared to the experimental data. Additionally, a lack of effect of the shear force between the wheel and soil in the preliminary model considered, the shear force is possibly added to the predicted value. This suggested that the preliminary model needs to be carefully considered further.

Further investigation of the normal stress distribution of the locked-wheel will focus on extending these experiments to the different wheel sizes, vertical loads, and soil types. This investigation provides more detailed information and general knowledge about the variable of the distribution. Additionally, the shear force between the wheel surface and soil, which was neglected in this paper, will be considered by modifying the sensor system or experimental setup. To develop the estimation model of the resistance force for the locked-wheel, the model will be carefully considered, including the effect of the shear force between a wheel and the soil through even more conditions.

## Figures and Tables

**Table 1:** Wheel sensor system.

Description	Values
Sensor controller	EBS-001-1
Amplifier board	AMF3-313103
Intervals angle (${}^{\circ }$)	10
Wheel diameter (m)	0.17
Wheel width (m)	0.04

**Table 2:** Tactile sensor: Shokac Chip SP.

Description	Values
External form size (m)	0.0097 × 0.006
Sensing area diameter (m)	0.0055
Force range vertical force $F_{z}$ (N)	+2
Force range shear force $F_{x}$, $F_{y}$ (N)	±2

**Table 3:** Configuration of sensor position $\theta_{e}$, $\theta_{o}$, as shown in Figure 10.

Case	$\theta_{e}$(${}^{\circ }$)	$\theta_{o}$(${}^{\circ }$)
A	−25	25
B	−20	30
C	−15	35
D	−10	40

**Table 4:** Relationship between smallest contact angle $\theta_{e}$ (${}^{\circ }$) and sinkage.

Sinkage (m)	Smallest Contact Angle $\theta_{e}$ (${}^{\circ }$)
0.013	−20
0.015	−15
0.018	−10
0.024	−5
0.027	0
0.033	0

**Table 5:** The smallest contact angle at each sinkage level measured by PIV analysis.

Sinkage (m)	Smallest Contact Angle (${}^{\circ }$)
0.012	−23.6
0.016	−14.5
0.018	−16.9
0.022	−6.9
0.028	0
0.033	0

**Table 6:** Soil parameters and values for calculation of resistance force.

Modulus	Value	Unit	Name of Parameters	Reference
-	Soil	-	Silica Sand No. 5	-
*v*	0.0003.41	(m/s)	Towing speed	-
*D*	0.17	(m)	Wheel diameter	-
$b_{w}$	0.04	(m)	Wheel width	-
*m*	2.5	(kg)	Wheel mass	-
*c*	762	$\left(N/m^{2}\right)$	Soil cohesion	[37]
$C_{a}$	762	$\left(N/m^{2}\right)$	Soil-tool adhesion	[37]
*g*	9.81	($m/s^{2}$)	Earth gravity	-
*q*	Measured	$\left(N/m^{2}\right)$	Surcharge on the soil surface	Measured by experiment
δ	15	${(}^{\circ })$	External friction angle	Decided by plate experiment
β	90	${(}^{\circ })$	Rake angle	-
γ	1430	($\mathrm{kg}/m^{3}$)	Soil density	Measured by experiment
κ	0.002–0.02	(m)	Shear deformation modulus	[37]
ϕ	22.3	(${}^{\circ }$)	Internal friction angle	[37]

## Appendix A. List of Major Notation

Table A1 summarizes the major notation used in this paper.

**Table A1:** List of notation.

Description (Unit)	Symbol
Wheel or plate width (m)	$b_{w},b_{p}$
Cohesion of the soil $\left(N/m^{2}\right)$	*c*
Soil-tool adhesion $\left(N/m^{2}\right)$	$C_{a}$
Height of the each block (m)	$d_{i}$
Diameter of the wheel (m)	*D*
Total resistance force of the locked-wheel (N)	$F_{t}$
Force of soil mass area B (N)	$F_{1}$
Shear force beneath the soil wedge area *A* (N)	$F_{2}$
Side friction force of the soil wedge area *A* (N)	$F_{3}$
Side friction force of the side surface of the wheel (N)	$F_{4}$
Shear force between wheel and soil (N)	$F_{5}$
Side friction force of the side surface at each block (N)	$F_{side_{i}}$
Total force acting on the area *B* (N)	$F_{M}$
Horizontal component of the total force (N)	$F_{Mh}$
Vertical component of the total force (N)	$F_{Mv}$
Earth gravity $\left(m/s^{2}\right)$	*g*
Shear displacement (m)	*j*
Coefficient of earth pressure at rest (−)	$K_{0}$
Soil modulus of deformation depend on c ($N/m^{\left(n+1\right)}$)	$k_{c}$
Internal friction angle modulus ($N/m^{\left(n+2\right)}$)	$k_{\phi }$
Width of the each block (m)	$l_{i}$
Pressure-sinkage ratio (−)	*n*
Soil pressure $\left(N/m^{2}\right)$	*p*
Surcharge on the soil surface $\left(N/m^{2}\right)$	*q*
Radius of wheel (m)	*r*
Object speed (m/s)	*v*
Weight of the plate (N)	$W_{p}$
Weight of the soil wedge (N)	$W_{s}$
Weight of soil at each block (N)	$W_{si}$
Weight of the wheel (N)	$W_{w}$
Weight of the wheel at each block (N)	$W_{wi}$
Sinkage (m)	$z,z_{0}$
Length of the surface between areas *A* and *B* (m)	$z_{v}$
Inclination angle of the side friction force and tangential force ${(}^{\circ })$	$\alpha_{i}$
Rake angle ${(}^{\circ })$	β
Soil density $\left(\mathrm{kg}/m^{3}\right)$	γ
External friction angle ${(}^{\circ })$	δ
Sensor position of the wheel sensor ${(}^{\circ })$	$\theta_{e}$, $\theta_{o}$
Static contact angle ${(}^{\circ })$	$\theta_{s}$
Arbitrary position of slip surface ${(}^{\circ })$	$\theta_{l}$, $\theta_{li}$
Shear deformation modulus (m)	κ
Shear plane failure angle ${(}^{\circ })$	ρ
Horizontal normal stress at each block $\left(N/m^{2}\right)$	$\sigma_{hi}$
Wheel weight per unit area at each block $\left(N/m^{2}\right)$	$\sigma_{wi}$
Vertical normal stress at each block $\left(N/m^{2}\right)$	$\sigma_{vi}$
Shear force beneath each block (N)	$\tau_{i}$
Internal friction angle ${(}^{\circ })$	ϕ
